# Treatment of gastropleural fistula with combined endoscopic therapy: endoscopic vacuum therapy and over-the-scope clip

**DOI:** 10.1016/j.vgie.2026.02.009

**Published:** 2026-03-03

**Authors:** Gustavo de Carvalho Bertaccini Guriam, Thiago de Castro Mendonça Furtado, Rodrigo Chini, Eduardo Garcia Pacheco, Arlindo Moi, Gustavo Murad Pinton, José Antônio Mansur Mendes, Jose Ricardo Cunha Neves, Leticia Pires Dutra

**Affiliations:** 1 Department of Endoscopy, São Francisco Hapvida Hospital, Ribeirão Preto, São Paulo, Brazil; 2 Department of General Surgery, São Francisco Hapvida Hospital, Ribeirão Preto, São Paulo, Brazil; 3 Universidade Cidade de São Paulo (UNICID, City University of São Paulo), São Paulo, Brazil

## Abstract

**Background and Aims:**

Gastropleural fistulas (GPFs) are rare pathologic communications between the stomach and the pleural cavity and are associated with high morbidity and limited success with conventional surgical repair. We aimed to describe the successful management of a complex, refractory GPF using a multimodal endoscopic approach.

**Methods:**

A 27-year-old man with traumatic diaphragmatic rupture and multiple failed surgical repairs was referred to our tertiary endoscopy center. Endoscopic evaluation confirmed a gastric-to-pleural fistula. A sequential strategy was used: initial endoscopic negative pressure therapy using a modified nasogastric vacuum system, followed by closure with an over-the-scope (OTS) clip.

**Results:**

Negative pressure therapy promoted granulation tissue formation and reduction of fistula output. Deployment of an OTS clip achieved complete sealing of the orifice, with no further thoracic drainage. Total endoscopic therapy lasted 28 days. The patient was discharged in good clinical condition. At 6-month follow-up, he remained asymptomatic, with endoscopy confirming a well-healed scar and no recurrence.

**Conclusions:**

Multimodal endoscopic therapy combining negative pressure therapy and OTS clip placement represents a safe and minimally invasive option for complex GPFs refractory to surgery. This case highlights the growing role of advanced endoscopic techniques in the management of challenging gastrointestinal fistulas.

## Introduction

Gastropleural fistulas (GPFs) are rare pathologic communications between the stomach and the pleural cavity and are associated with high morbidity and potentially life-threatening outcomes.^1^ They pose a significant therapeutic challenge, particularly in complex cases in which conventional approaches such as surgery may fail to achieve closure or lead to postoperative adverse events.

Given their rarity, no standardized treatment protocol has been established. Historically, open surgical repair via laparotomy was the preferred option, with thoracotomy reserved for cases of extensive thoracic contamination.^2^ More recently, advances in endoscopic techniques have expanded the therapeutic armamentarium for gastrointestinal fistulas, providing less-invasive alternatives associated with lower adverse event rates and improved outcomes.^3^, ^4^, ^5^ Endoscopic modalities, including debridement, internal drainage with pigtail stents, and negative pressure therapy, have shown efficacy in reducing fistula-related morbidity and mortality.^6^, ^7^, ^8^ Although each technique has limitations when applied in isolation, their combination has demonstrated promising results, especially in complex cases requiring a multimodal approach.

## Case description

We report the case of a 27-year-old man with a history of motorcycle trauma resulting in diaphragmatic rupture, gastric herniation, and a subsequent GPF (Video 1, available online at www.videogie.org). The fistula had been surgically addressed on 4 separate occasions without success, including 2 laparotomies with attempted direct repair, 1 thoracotomy, and ultimately esophagostomy and gastrostomy for diversion, plus jejunostomy for nutritional support. The patient was referred to our tertiary endoscopy center approximately 45 days after the initial injury.

Prereferral CT confirmed herniation of the gastric fundus, with a tube traversing the esophagostomy and reaching the gastric body. A left chest drain remained in place, with residual pleural effusion and pneumothorax associated with near-complete collapse of the left lung. Gastrostomy and jejunostomy tubes were also present. Finally, careful review of the imaging showed no sizable vessels near the suspected fistula tract or within the planned endoscopic vacuum therapy (EVT) contact area.

**Figure 1:**
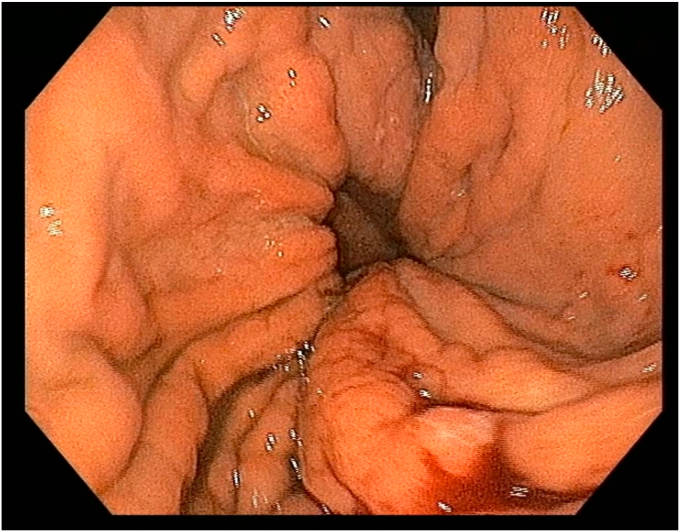
Herniation of the gastric fundus.

**Figure 2:**
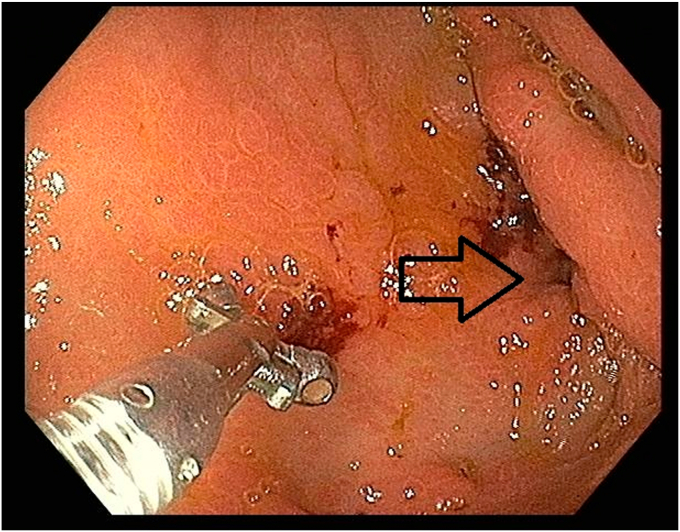
Gastropleural fistula indicated by the *arrow*.

Despite the severity of the patient's condition, he remained hemodynamically stable but presented with high-output bilious drainage from the left thoracic drain (750 mL/24 h). Endoscopic evaluation was performed through the esophagostomy orifice, and conservative management was initiated using a gastroscope (Olympus GIF-Q170, Tokyo, Japan) with carbon dioxide insufflation. Endoscopy demonstrated gastric fundus herniation above the level of the diaphragmatic rupture and a 3-mm perforation near the angle of His with bilious drainage (Figs. 1 and 2).

**Figure 3:**
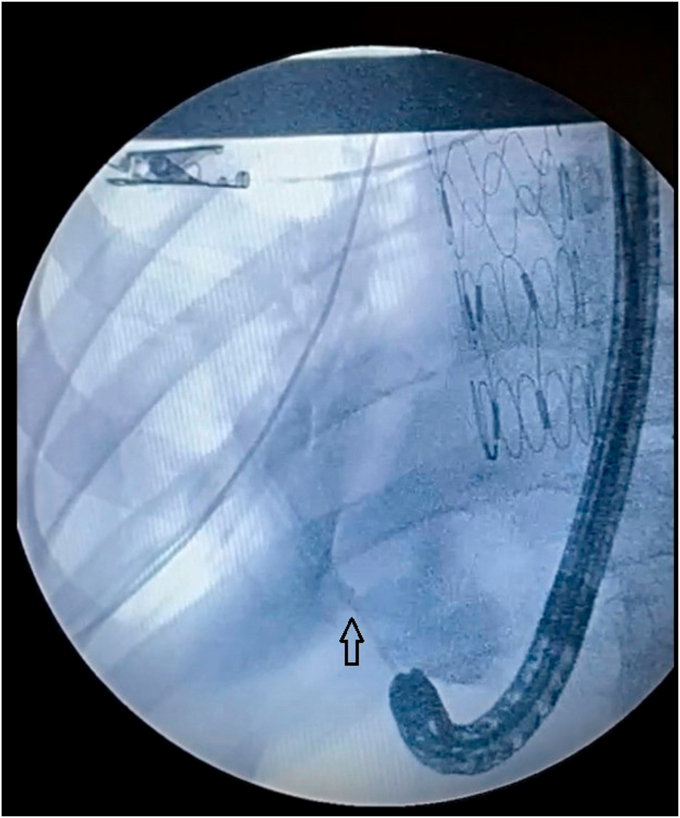
Gastropleural communication highlighted by the *arrow*.

During the same session, contrast injection through the approximately 3-mm gastric orifice outlined a linear, 12-mm fistulous tract communicating the gastric lumen with the pleural cavity. We then irrigated the pleural cavity with saline solution through the gastric orifice, confirming direct gastropleural communication (Fig. 3).

**Figure 4:**
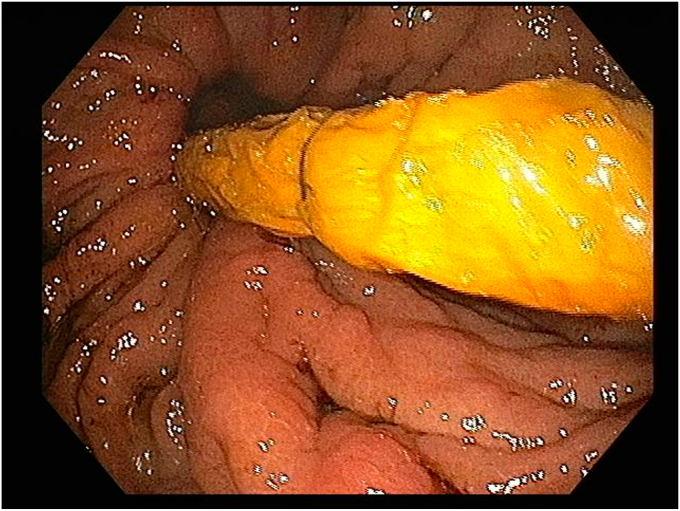
Modified endoscopic vacuum suction nasogastric drain.

Given the chronicity of the fistula and concern for a persistent, potentially epithelialized tract, we elected a staged endoscopic strategy. We first used EVT^6^^,^^8^^,^^9^ to achieve continuous internal drainage, reduce local contamination, and stimulate granulation tissue formation, thereby promoting progressive collapse of the fistulous channel and control of the associated cavity. After adequate tract “conditioning” and cavity control, we proceeded with definitive mechanical closure. A modified vacuum-assisted nasogastric drain^9^ was positioned endoscopically adjacent to the fistula and secured with endoscopic clips, allowing targeted application of continuous negative pressure at −125 mm Hg (Fig. 4). The EVT system was exchanged at 14-day intervals, consistent with our protocol and prior reports.^8^

**Figure 5:**
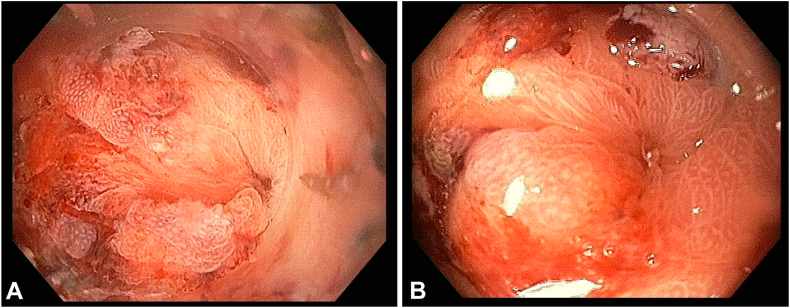
**A**, Granulation tissue with local friability. **B**, Decrease in the size of the fistula opening.

After 14 days of therapy, the thoracic drain output decreased to 250 mL/d. Endoscopic reevaluation revealed granulation tissue at the fistula site and a reduction in fistula size (Fig. 5A and B). To optimize closure, an over-the-scope (OTS) clip was deployed, resulting in complete sealing of the orifice.^7^^,^^8^^,^^10^^,^^11^ Vacuum therapy was continued for another 14 days in parallel with the clip to overcome technical challenges in securing fibrotic mucosa and to mitigate the risk of early clip migration, while also maintaining the healing stimulus through negative pressure.

**Figure 6:**
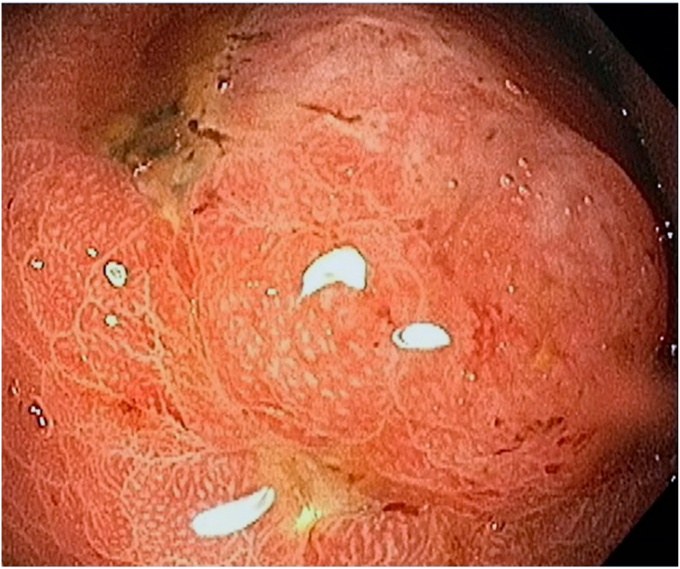
Correct positioning of the over-the-scope clip was confirmed, and no fistula orifice was identified after 14 days of combined endoscopic treatment.

No thoracic drain output was observed from the first day after clip placement. After 14 consecutive days without drainage and 28 days of total endoscopic therapy, a third endoscopy confirmed stable clip position and absence of a fistulous opening (Fig. 6). The treatment was deemed successful, and the patient was transferred back to the referring hospital for reversal of the esophagostomy, gastrostomy, and jejunostomy and for removal of the thoracic drain.

**Figure 7:**
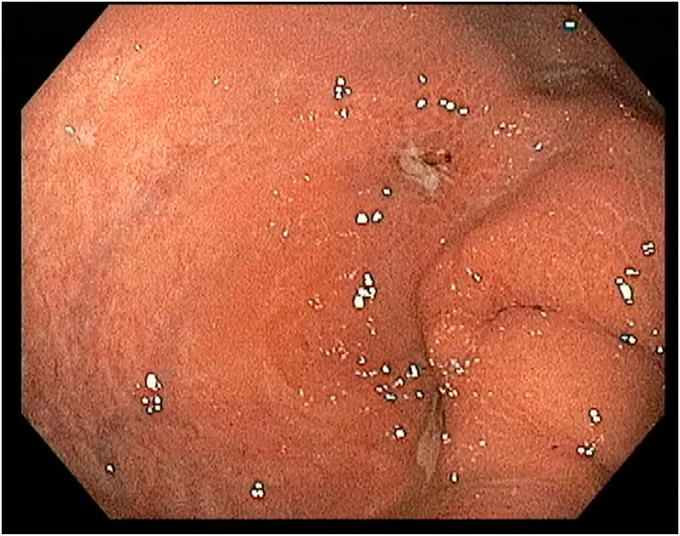
Scar tissue in the region of the previous fistula, with no presence of the over-the-scope clip, during long-term follow-up.

At 6-month follow-up after discharge, the fistula remained closed. The patient was asymptomatic and in good health. Surveillance endoscopy revealed a well-healed scar without recurrence or a retained clip (Fig. 7). The OTS clip had detached spontaneously and was naturally expelled in the stool.

Follow-up chest CT demonstrated a posteromedial diaphragmatic hernia containing fat and the gastric fundus, with mild compression of the adjacent lung and associated atelectasis. Importantly, there was no CT evidence of a persistent GPF or adjacent collections. A small laminar left pleural effusion and mild posterior basal consolidation were also noted.

## Discussion

This case underscores the value of a sequential multimodal endoscopic approach for managing a complex GPF refractory to multiple surgical attempts. It highlights the growing role of endoscopic management for transmural gastrointestinal and gastropleural defects, particularly when surgery carries high risk or limited success.

Negative pressure therapy was effective in stimulating granulation tissue and controlling local infection, aligning with prior evidence for its use in gastrointestinal fistulas and cavity collections.^6^^,^^12^ The OTS clip, used as the final step, ensured definitive closure and minimized the need for further invasive interventions, enabling safe discharge.

## Conclusion

Multimodal endoscopic therapy can provide an effective, minimally invasive solution for complex fistulas. This case illustrates the clinical utility of combining advanced endoscopic techniques in gastroenterology and supports their role in the management of challenging gastrointestinal conditions.

## Disclosure

All authors disclosed no financial relationships.

## Supplementary data

Video 1 xxx
