# Supplementary figures and images for: Treatment of gastropleural fistula with combined endoscopic therapy: endoscopic vacuum therapy and over-the-scope clip
Source: VideoGIE. 2026 Mar 3;11(7):275–8. doi: 10.1016/j.vgie.2026.02.009 (PMC13343435; doi:10.1016/j.vgie.2026.02.009)

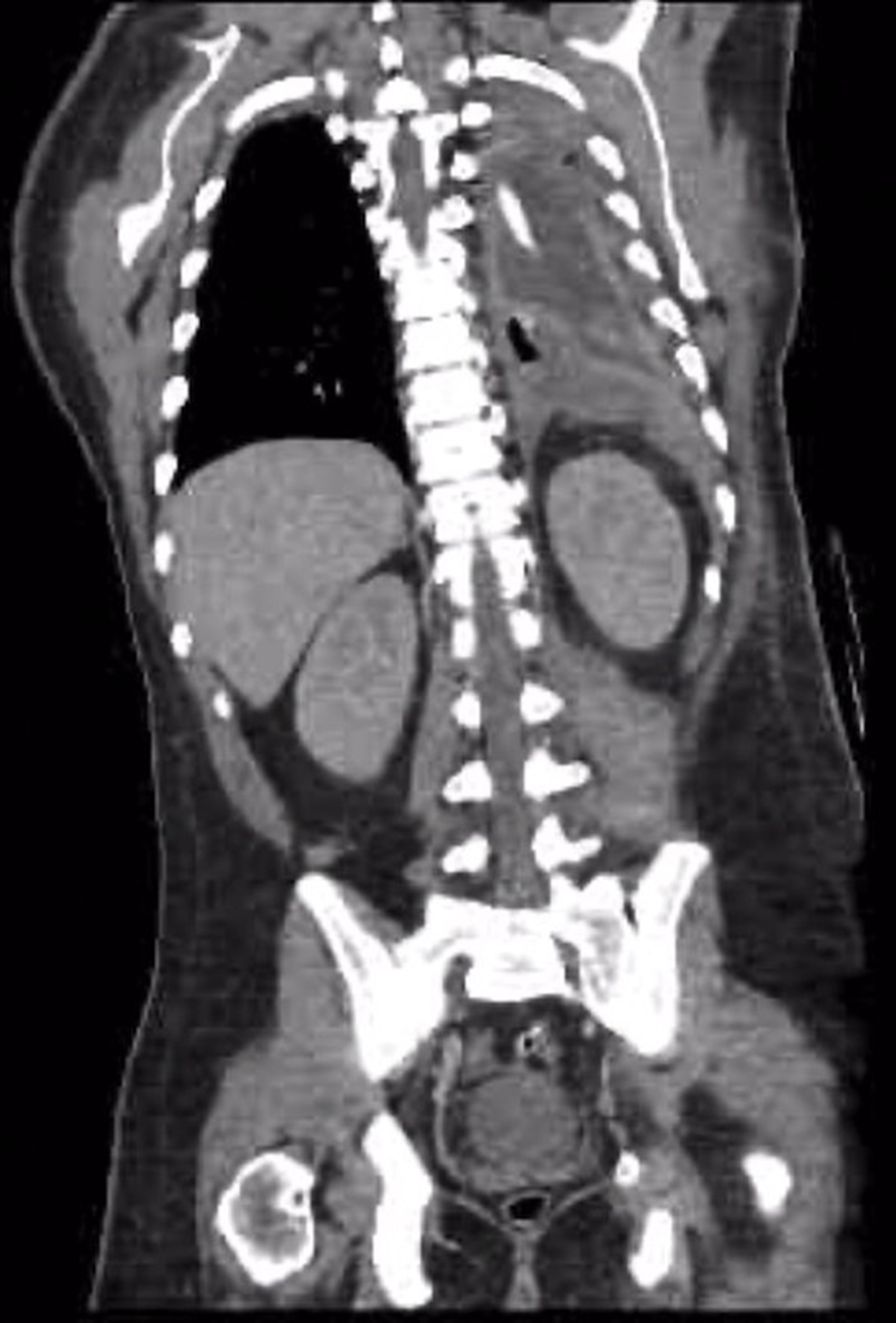

Supplement: Supplementary Figure [file figs1.jpg]
